# Small-Pox at the Perth Infirmary

**Published:** 1887-10-29

**Authors:** David H. Stirling


### Small-pox at the Perth Infirmary.

By David H. Stirling, M.D.

My attention has been called to your article, "Medical Scien at Perth," and I have to ask you to allow me space to reply to your remarks. As yours is in part a medical journal, a somewhat detailed statement may be permissible.

The Perth Infirmary consists of three blocks, quite apart from each other, one of these specially built for infectious diseases in 1865. Up to 1876 small-pox was received and treated in the Infirmary. At that time it was decided that small-pox should be no longer admitted, and an appeal from the Local Authority to withdraw this restriction was rejected. Since that time there has been practically no small-pox in this locality. When the Infirmary Directors decided to refuse admission to small-pox patients (my name is one of those signing the letter requesting that it should be so), the Local Authority proceeded to erect a hospital for infectious diseases at a distance from human dwellings. Two or three times since then one or two patients have been under treatment there. There is a male and female ward in the hospital, each, I understand, capable of receiving from six to eight patients,—as to this I am not quite certain. The forthcoming report of Dr. Littlejohn, of Edinburgh, who has been here to-day, may give us some particulars about that hospital.

On October 4th I was asked to see three female patients lying in the central block, in General Ward No. 1, as they were suspected to be cases of small-pox. There was no doubt as to this, and immediate removal was an urgent necessity. They were taken to the small-pox hospital the following night—the hospital not being ready to receive patients at once. The nurse who accompanied them looked at the place, and saw a scene of utter unpreparedness to receive them even then. Three beds were, so far, ready in one ward; in the other, cleaning and repairs were in progress. She was asked to be particular in her observations, as three of the nurses were showing suspicious symptoms, as well as another female patient. Early next day the disease was unmistakable, but with them I had nothing to do. The acting medical officers having heard of the state of matters at the small-pox hospital, in consultation with the matron, saw that it would be easy to set apart the west wing for all small-pox cases that might arise in connection with the unfortunate accident, as it may be called, and scrupulous precautions in removal and in disinfection of every infected place and thing were taken. The following morning I was informed of what had been done, and with it all I fully agreed. One other nurse, who had assisted in removing the three nurses and fourth female patient attacked, sickened, about eight days later, and may have had small-pox, but certainly in the most modified form. She was also removed to the west wing, a large, admirably ventilated building, quite detached from every other. General revaccination was performed over the whole house as soon after the first outbreak as possible. The female patient attacked was suffering from severe cardiac affection, and died, vaccima and variola running on together.

On the 8th a special meeting of Directors was called—several of the Local Authority are ex-officio members. I was asked as to the safety of the other inmates of the Infirmary in the circumstances, and replied, that from the precautions taken I was satisfied that no harm would result. Special nurses had been got from Edinburgh, rigid isolation was enforced, every disinfecting precaution was carried out, and in fact nothing more could be done to ensure safety. I held then, and hold still, that the best course possible was adopted. If this unhappy occurrence, and the outcry made by certain parties in the town, lead to the Directors of the Infirmary refusing admission to all infectious cases, the ratepayers will know on whose shoulders the blame lies, or to whom praise is due. Perth is heavily burdened now; whether another straw will not break its back will be known when the new load is attempted to be laid on. Should the necessity arise, the Local Authority will require to provide a very different hospital and staff from that which they now possess.

I am not responsible for expressions used by others, and the phrase "no scintilla of danger," put in my mouth by the Lord Provost, is new to me. I can hardly suppose that any respectable medical journal would write of any medical man as you have done of me in the article I now reply to, without having a foundation of supposed fact, resting on hitherto trusted authority. This being so, I must say further, that your article bears traces of more than zeal for truth in science, and in many ways points to a local origin of a very questionable kind. It is fiction, not fact; falsehood, not truth.

Your little note as to Miss Logan's share in the matter will be estimated at its true value here. The assertions therein contained have been proved to be utterly untrue—assertions made by a public official on hearsay evidence, cruelly injurious to the reputation of one who has proved her fitness for her duties by long and faithful service.

Daily papers in Dundee and local journals have been supplied with similar material to that which you produce, and even after a correct version of the whole history of small-pox in relation to Infirmary management has been sent to and inserted by one of those papers, the tone of letter-writing in the anonymous correspondence and in "Editorials" remains as before. The correctness of my statements, however, may be verified by reference to the minutes of the Directors. I shall inform you when the report of Dr. Littlejohn appears, and, if possible, forward a copy of it. Meantime I trust to your inserting this letter in next issue.

*** We gladly insert Dr. Stirling's letter. He and others may be assured, however, that the article in question was not in any way locally "inspired" or written, but was based entirely on the report in the *Dundee Advertiser*. The writer of the article is not personally acquainted with any medical man in Perth or Dundee, and is not even a Scotchman. We give this explanation in order that there may be no local misunderstandings.—[Ed. T. H.]

